# VARIATION IN ADVANCE DIRECTIVES TIMING BETWEEN END-STAGE RENAL DISEASE PATIENTS AND CANCER PATIENTS

**DOI:** 10.1093/geroni/igz038.1018

**Published:** 2019-11-08

**Authors:** Yu-Hsuan Wang, Susan Enguidanos

**Affiliations:** 1 Leonard Davis School of Gerontology, University of Southern California, Los Angeles, California, United States; 2 University of Southern California, Los Angeles, California, United States

## Abstract

The United States has the third highest prevalence and the second highest incidence of End-Stage Renal Disease (ESRD). ESRD is associated with high mortality and lower quality of end-of-life experiences. Having an advance directive (AD) is associated with better care at the end of life. Although past ACP completion rates in ESRD patients has been studied, little is known about its timing differences between ESRD and cancer patients. This study investigates the timing difference of AD completion between ESRD and cancer patients We conducted logistic regression to analyze data from the Health and Retirement Study, a nationally representative longitudinal survey of older adults. The analytic sample included exit interviews from 2012 to 2016 among 971 proxies of deceased with ESRD or cancer. Among the sample, 47% of decedents completed an AD; 44% of cancer patients and 48% of ESRD patients. Being a racial minority (OR=0.38, p<0.001), and lower education (OR= 0.63, p=0.001) were associated with lower AD completion rates. No significant differences in AD completion rates were found between cancer patients and ESRD patients. Compared to cancer patients, ESRD patients were more likely to complete ADs more than one year before death (OR=3.15, p=0.001). However, there were no significant difference between cancer patients and ESRD patients in AD completion rates in the three months before death. Although both samples had comparable rates of AD completion, compared to cancer patients, ESRD patients tend to document care preferences earlier. Further studies are needed to investigate factors related to early documentation of ADs.

